# Neuropeptide Y regulates proliferation and apoptosis in granulosa cells in a follicular stage-dependent manner

**DOI:** 10.1186/s13048-019-0608-z

**Published:** 2020-01-08

**Authors:** Yoko Urata, Reza Salehi, Patricia D. A. Lima, Yutaka Osuga, Benjamin K. Tsang

**Affiliations:** 1 Departments of Obstetrics & Gynecology and Cellular & Molecular Medicine, Interdisciplinary School of Health Sciences, University of Ottawa; Chronic Disease Program, Ottawa Hospital Research Institute, Critical Care Wing, 3rd floor, Room W3107, 501 Smyth Road, Ottawa, ON K1H 8L6 Canada; 2 Department of Obstetrics and Gynecology, the University of Tokyo, 7-3-1 Hongo, Bunkyo-ku, Tokyo, 113-8655 Japan; 3 Queen’s Cardiopulmonary Unit, Queen’s University, BioSciences Complex, Room 1605, 116 Barrie Street, Kingston, ON K7L 3N6 Canada

**Keywords:** Neuropeptide Y, Folliculogenesis, Neuropeptide, Proliferation, Apoptosis

## Abstract

**Background:**

The complex regulatory mechanism involved in ovarian follicular development is not completely understood. Neuronal neuropeptide Y (NPY) is involved in the regulation of feeding behavior, energy homeostasis, and reproduction behavior, while its function in ovarian follicular development is not clear. The objective of this study was to investigate if and how NPY regulates follicle development in the ovary.

**Methods:**

All experiments were performed using Sprague Dawley rats. To understand NPY expression pattern at different stages of follicular development, NPY content was assessed using immunohistochemistry in individual follicles. NPY and its receptors expression pattern were evaluated in granulosa cells isolated from preantral (PA), early antral (EA) and late antral follicles (LAF). The influence of NPY on granulosa cell proliferation and apoptosis were further assessed in vitro*,* using Ki67- and TUNEL-positivity assays. To investigate whether NPY induced-proliferation in EA granulosa cells is mediated through the activation of NPY receptor Y5 (NPY5R) and Mitogen-activated protein kinase (MEK) signal pathway, EA granulosa cells were treated with NPY5R antagonist (CGP71683) and MEK inhibitors (PD98059 and U0126), and Ki67-positive cells were assessed.

**Results:**

NPY protein expression was follicular stage-dependent and cell type-specific. NPY signal intensity in EA was higher than those in PA and LAF. Antral granulosa cells showed the highest signal intensity compared to mural granulosa cells, cumulus cells and theca cells. Granulosa cells NPY protein content and mRNA abundance were higher in EA than in LAF. NPY receptor contents in granulosa cells were follicular stage-dependent. While NPY reduced apoptosis of EA granulosa cells, it increased the proliferation through NPY5R and MEK pathway. In contrast, in LAF granulosa cells, NPY reduced proliferation and increased the number of apoptotic cells, with no significant effects on PA granulosa cells.

**Conclusion:**

This study is the first to evaluate the intraovarian role of NPY in granulosa cells at various stage of follicular development. These results indicate that NPY regulates granulosa cells proliferation and apoptosis in a follicular stage-dependent and autocrine manner. NPY may play a role in pathogenesis of ovarian follicular disorders.

## Background

Neuropepetide Y (NPY), a 36 amino-acid neuropeptide, is abundantly expressed in central and peripheral nervous system [1]. Five mammalian NPY receptors (Y1, Y2, Y4, Y5 and y6) have been cloned. All of Y1, Y2, Y4 and Y5 receptors are coupled to an inhibitory G protein, except the y6 receptor which is truncated in the most mammals including human, but is functional in mice [2]. Neuronal NPY is involved in the regulation of feeding behavior [3], energy homeostasis [4], memory retention [5] and reproductive behavior [6]. Additionally, it has been reported that NPY also regulates proliferation [7, 8], apoptosis [9], immune [10] and reproductive functions as it is expressed by leydig cells [7, 11]. Moreover, NPY acts on the luteal vascular system regulating corpus luteum function [8]. However, it is not clear whether and how NPY regulates follicle development.

In the ovary, NPY is expressed in the stromal tissue of small antral follicles, granulosa cells of large antral follicles as well as in the nerves and vessels of the theca internal layer [8, 9]. Jørgensen et al. reported that ovarian follicular fluid contains NPY [10]. Moreover, NPY regulates the luteal vascular system in the sheep and induces oxytocin secretion from the corpus luteum [8], stimulates luteal estradiol synthesis and granulosa cell progesterone secretion in vitro [12, 13]. It induces Bcl-2-associated X (BAX) mRNA and decreases proliferating cell nuclear antigen (PCNA) mRNA in porcine luteinized granulosa cells [14]. However, the main ovarian source of NPY, and whether and how NPY regulates follicular growth are not known.

The objectives of this study were to investigate (a) whether the granulosa cell is the follicular source of NPY; (b) if and how NPY regulates granulosa cell proliferation and follicular atresia; (c) which of the NPY receptors mediate its actions on the granulosa cell proliferation and apoptosis; and (d) whether the above mechanisms are follicular stage-specific. We hypothesized that the pattern of NPY and its receptor expression can determine granulosa cell fate (proliferation vs apoptosis) and is follicular stage-dependent. To test this hypothesis, we assessed the expression of NPY and its receptors in granulosa cells isolated at different ovarian follicular stages in vivo. We also investigated the influence of NPY on granulosa cell proliferation and apoptosis at different follicular stages of development in vitro, as well as the signaling pathway involved in NPY-induced proliferation.

## Results

### Ovarian NPY mRNA and protein content is cell type- and follicular stage-specific

**Fig. 1:**
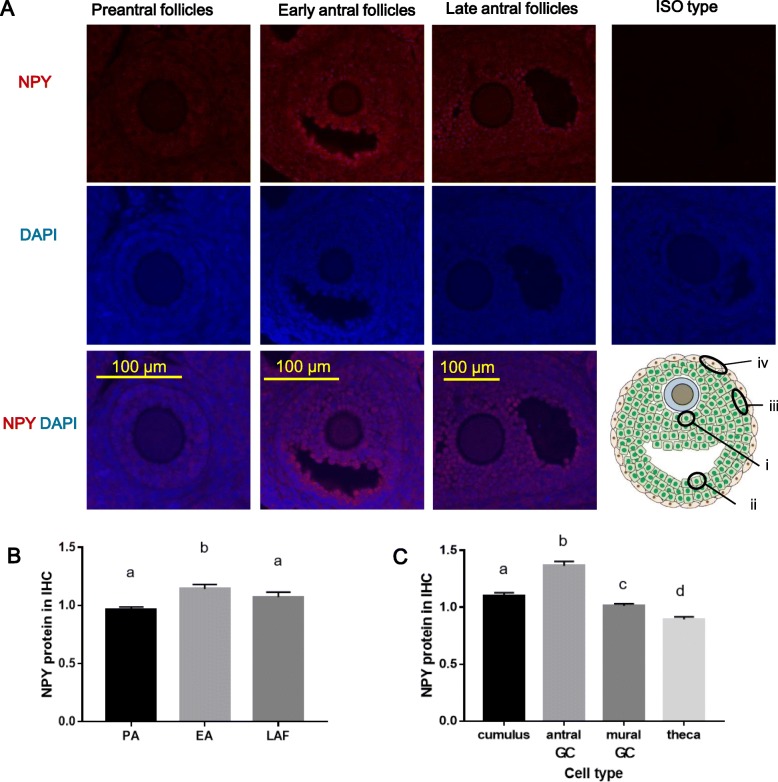
NPY expression is follicular stage-dependent and antral GC is the main ovarian follicular source. Immunofluorescence of NPY in ovaries was performed using 21-day-old rat. **a** Representative pictures are shown. **b, c** The signal intensity of NPY in single cells was measured; cumulus cells (cumulus; A, i), antral granulosa cells (antral GC; A, ii), mural granulosa cells (mural GC; A, iii) and theca cells (theca; A, iv) in preantral, early antral and late antral follicles (PA, EA and LAF, respectively). NPY signal intensity in EA was higher than that in PA and LAF **b**, and that of antral GC was higher than that of cumulus, mural GC and theca **c**. These results suggest that NPY expression is follicular stage-dependent and antral GC is the main follicular source. Data are representative of the mean ± SEM using six biological replicates and analyzed by one-way ANOVA and Tukey’s multiple comparison. Different letters denote significant differences in Tukey’s multiple comparison (*p* < 0.05)

To determine if the granulosa cell is the primary follicular source of NPY, and whether its expression is follicular stage-specific, NPY signal intensity and localization were assessed by immunofluorescence in 21-day-old Sprague Dawley (SD) rat ovaries. NPY signals were evident in granulosa cells in primordial (data not shown), preantral (PA), early antral (EA) and late antral follicles (LAF), but could not be demonstrated in atretic follicles due to non-specific binding (data not shown). NPY signal intensity in EA (one or multiple small fluid-filled cavities) was significantly higher than those in LAF (one fluid-filled cavity) and PA (*p* < 0.05). There was no significant difference in its signal intensity between PA and LAF (Fig. 1b; *p* > 0.05). To determine if its expression is cell type-specific, we assessed the signal intensities of cumulus (granulosa cells beside the oocyte, Fig. 1a, i), antral (granulosa cells beside the antrum, Fig. 1a, ii) and mural granulosa cells (granulosa cells beside the basal lamina, Fig. 1a, iii) and theca cells (Fig. 1)a, iv. Since antral granulosa cells had a higher NPY signal intensity than cumulus, mural granulosa and theca cells (Fig. 1c; *p* < 0.05), we focused our subsequent experiments on NPY in antral granulosa cells from EA stage. These results suggest that granulosa cell is the main follicle source of NPY and its expression is follicular stage-dependent.

**Fig. 2:**
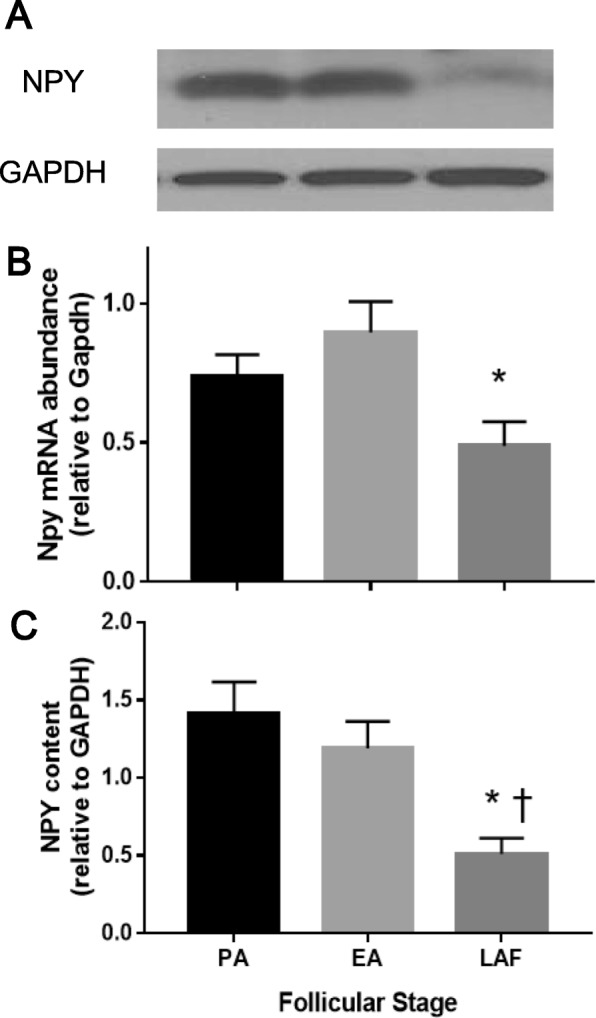
NPY mRNA abundance and protein content of granulosa cells is higher in EA than LAF. Granulosa cells of PA, EA and LAF were isolated separately using rats primed with eCG, as described in the methods section. **a, c** Total protein was extracted and NPY contents were assessed by Western blot. Representative Western blot result is shown **a**. NPY protein content of granulosa cells in PA and EA was higher than that in LAF **c**. **b** Total mRNA was extracted, reverse transcribed and amplified by real-time PCR using specific primers for Npy and Gapdh. Expression of Npy mRNA was normalized to RNA loading for each sample using Gapdh mRNA as an internal standard. Npy mRNA of granulosa cells in EA was higher than that in LAF. These results suggest that granulosa cell NPY is more important for pre-antral and early-antral follicles development than at later stages. Each granulosa cell replicate was isolated from at least two rats. Results are expressed as mean ± SEM of independent replicates (A, *n* = 4; B, *n* = 5) and analyzed by one-way ANOVA and Tukey’s multiple comparison. *, *p* < 0.05 (vs. EA); †, *p* < 0.05 (vs. PA)

To further confirm if granulosa cells express NPY in a follicular stage-dependent manner, Npy mRNA abundance and protein content were assessed in granulosa cells isolated from different follicular stages following in vivo stimulation with equine Chorionic Gonadotropin (eCG) for 0 (PA), 24 (EA) or 48 h (LAF). Npy mRNA abundance in granulosa cells of EA was 1.8 times higher than that of LAF (Fig. 2b; *p* < 0.05) and that of PA tended to be higher than that of LAF (Fig. 2 b; *p* = 0.17). NPY protein content in granulosa cells of PA and EA were 2.7 and 2.3 times higher than that of LAF, respectively (Fig. 2a, c; *p* < 0.05), suggesting that granulosa cell NPY may be more important for the development of PA and EA than of LAF.

### Expression of granulosa cell NPY receptor is subtype-specific and dependent on the stage of follicular development

**Fig. 3:**
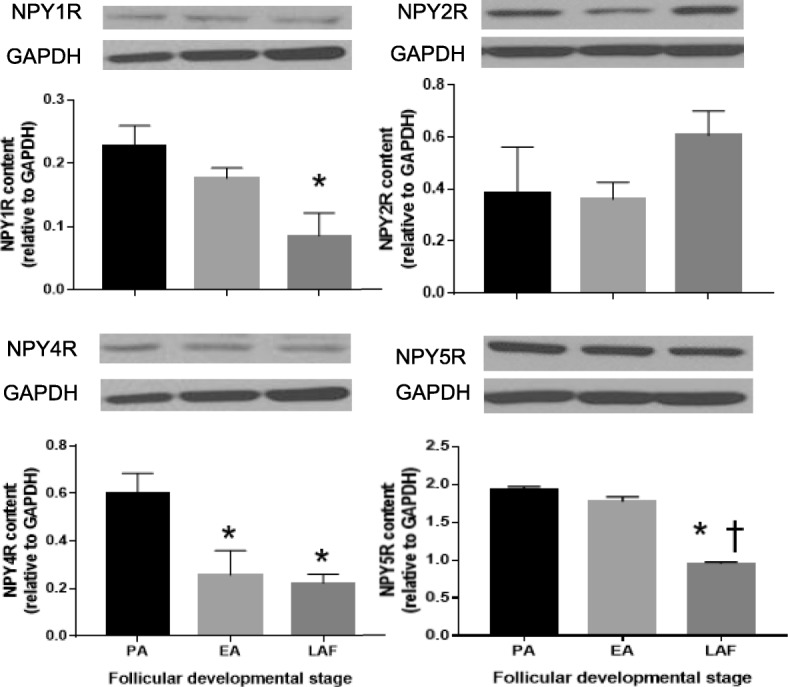
NPY receptor Y5 content of granulosa cells in EA is higher than that in LAF. Granulosa cells of PA, EA and LAF were isolated separately from rats primed with eCG, as described in the methods section. Total protein was extracted and NPY receptors contents were assessed by Western blot (*n* = 3). Representative Western blot results are shown. NPY receptor Y1 (NPY1R) content of granulosa cells in PA was higher than that in LAF. NPY receptor Y2 (NPY2R) contents of granulosa cells showed no significant difference. NPY receptor Y4 (NPY4R) content of granulosa cells in PA was higher than that of EA and LAF. NPY receptor Y5 (NPY5R) content of granulosa cells in PA and EA was higher than that in LAF. These results suggest that expression of NPY receptor is subtype-specific and may be involved in the regulation of granulosa cell functions in a follicular stage-dependent manner. Each granulosa cell replicate was isolated from at least two rats. Results are expressed as mean ± SEM of three independent replicates and analyzed by one-way ANOVA and Tukey’s multiple comparison. *, *p* < 0.05 (vs. PA); †, *p* < 0.05 (vs. EA)

To determine whether granulosa cell NPY receptors are expressed in a follicular stage-dependent manner, the protein content of various NPY receptor subtypes in granulosa cells from different stages of follicular development were compared. NPY receptor Y1 (NPY1R) content in granulosa cells of PA was 2.6 times higher than those of LAF, while NPY receptor Y2 (NPY2R) content in granulosa cells was not significantly different among the follicular stages. NPY receptor Y4 (NPY4R) content in granulosa cells of PA was 2.3 and 2.7 times higher than those of EA and LAF, respectively. NPY receptor Y5 (NPY5R) content in granulosa cells of PA and EA was 2.0 times and 1.9 times higher than those of LAF, respectively (Fig. 3). These results indicate that the expression of NPY receptor is subtype-specific and may be involved in the regulation of granulosa cell functions in a follicular stage-dependent manner.

### NPY induces proliferation in granulosa cells from early antral follicles and apoptosis from late antral follicles

**Fig. 4:**
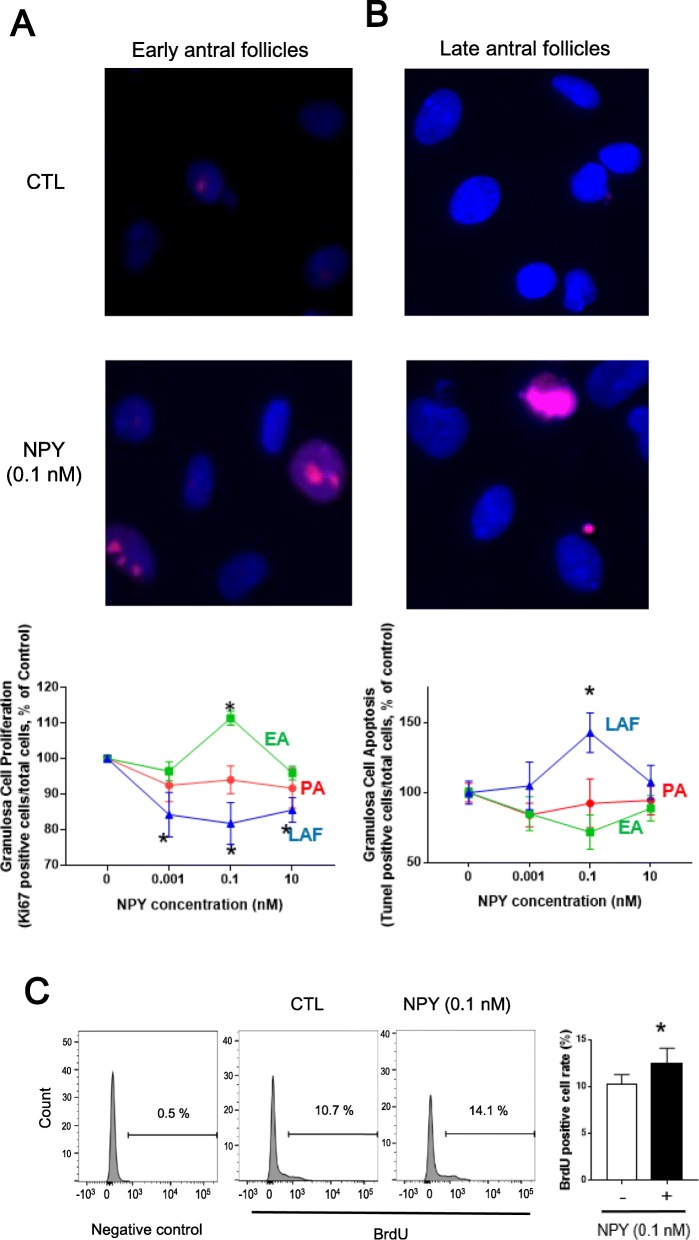
NPY induces granulosa cell proliferation in EA and reduced that in LAF. Granulosa cells of PA, EA and LAF were isolated separately using rats primed with eCG, as described in the methods section. **a, b** Granulosa cells were incubated with various concentration of NPY for 24 h and immunofluorescence of Ki67 (proliferation) and TUNEL assay (apoptosis) were performed. NPY induced Ki67-positivity of granulosa cells in EA (0.1 nM), while reduced that in LAF (0.001, 0.1, 10 nM; A). NPY induced TUNEL-positivity of granulosa cells in LAF (0.1 nM; B). **c** Granulosa cells from EA were incubated with NPY (0.1 nM) for 24 h and BrdU (10 μM) for last 4 h, and BrdU positive cells were detected by flow cytometry. Numbers displayed indicate percentage of single BrdU positive cells. NPY (0.1 nM) induced BrdU-positive granulosa cells in EA. These results suggest that NPY regulates granulosa cell proliferation and apoptosis in a follicular stage-dependent manner, inducing granulosa cell proliferation in early antral stage but apoptosis in late antral stage. Results are expressed as mean ± SEM of independent replicates (A, *n* = 5; B, *n* = 4; C, n = 4) and analyzed by two-way ANOVA and Tukey’s multiple comparison **a,b** and paired t-test **c**. *, *p* < 0.05 (vs. without NPY). Representative pictures and flow cytometry results are shown

To investigate if NPY regulates granulosa cell fate (proliferation and apoptosis) in a follicular-stage dependent manner, granulosa cells were isolated from ovarian follicles in different stages of development and they were cultured in the presence of NPY at various concentrations (0, 0.001, 0.1 and 10 nM). To determine the rate of proliferation and apoptosis, Ki67- and TUNEL-positivity were assessed, respectively. Our results indicate that NPY at 0.1 nM significantly increases Ki67-positivity in granulosa cells of EA by 11% (*p* < 0.05), while reduces those of LAF by 15.7, 18.2, 14.5% at 0.001, 0.1, 10 nM, respectively (Fig. 4a; *p* < 0.05). In contrast, NPY at 0.1 nM increases TUNEL-positivity in granulosa cells of LAF by 42% (Fig. 4b; *p* < 0.05). Additionally, NPY (0.1 nM) increased the number of BrdU-positive granulosa cells of early antral granulosa cells by 1.2-folds (Fig. 4c; *p* < 0.05). Our results indicate that NPY regulates granulosa cell fate in a follicular stage-dependent manner, inducing granulosa cell proliferation in early antral stage, but apoptosis in late antral stage.

### NPY induces granulosa cell proliferation through the activation of the NPY5R and via the mitogen-activated protein kinase pathway

**Fig. 5:**
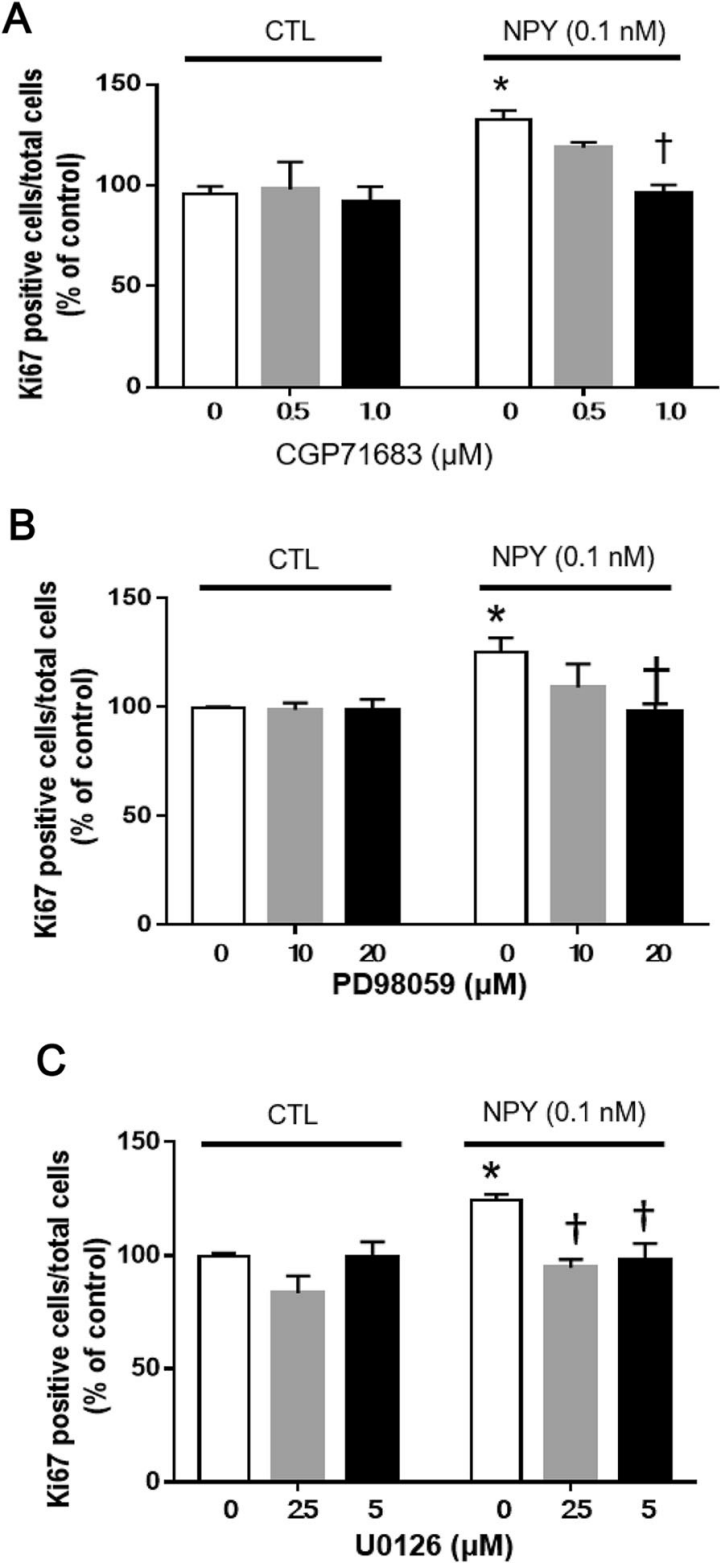
CGP71683, PD98059 and U0126 inhibited NPY-induced granulosa cell proliferation in early antral follicles. Granulosa cells in EA were pre-incubated with CGP71683 (NPY5R antagonist), PD98059 or U0126 (MEK inhibitors) for 1 h and stimulated with NPY (0.1 nM) for 24 h. Cell proliferation (Ki67-positivity) was assessed by immunocytochemistry. CGP71683, PD98059 or U0126 inhibited NPY-induced Ki67-positivity of granulosa cells. Each granulosa cell replicate was isolated from at least two rats. Results are expressed as mean ± SEM of independent replicates (A, n = 4; B, *n* = 5; C, *n* = 4) and analyzed by two-way ANOVA and Tukey’s multiple comparison. *, *p* < 0.05 (vs. without NPY and CGP71683/MEK inhibitors); †, *p* < 0.05 (vs. with NPY)

Since NPY5R contents of granulosa cells in EA was higher than that in LAF, we investigated whether NPY induced-proliferation is mediated through NPY5R activation by examining the influence of the NPY5R antagonist CGP71683 on NPY-induced granulosa cell proliferation. The NPY5R antagonist (1 μM) significantly inhibited NPY-induced-Ki67 positivity in granulosa cells of EA (Fig. 5a; *p* < 0.05). In the absence of exogenous NPY, CGP71683 (0.5, 1 μM) had no significant effects on Ki67-positivity and trypan blue positivity (cell viability marker; data not shown), suggesting that the effect of the antagonist was specific and not cytotoxic.

**Fig. 6:**
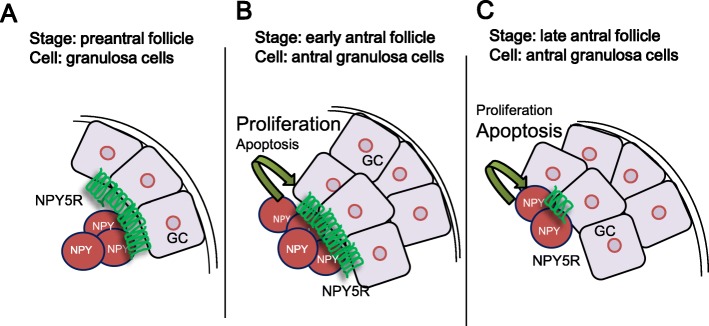
A hypothetical model illustrating NPY function in granulosa cells during the follicular development. **a** At preantral follicles, NPY plays minimal or no role in the regulation of proliferation nor apoptosis in granulosa cells. **b** At early antral follicles, NPY-secreted by granulosa cells induces proliferation via NPY5R in an autocrine manner. **c** At late antral follicles, both of NPY-secreted by granulosa cells and granulosa cell NPY5R expression decrease comparing to those at early antral follicles. NPY-secreted by granulosa cells induced apoptosis in an autocrine manner, which may involve other NPY receptor

To determine whether NPY-induced granulosa cell proliferation in EA is mediated through the Mitogen-activated protein kinase (MEK) signal pathway, granulosa cells were pre-treated for 1 h separately with the MEK inhibitors, PD98059 and U0126, and proliferation were assessed. Both of MEK inhibitors, PD98059 (20 μM) and U0126 (2.5 and 5 μM), significantly attenuated NPY-induced granulosa cell proliferation (Fig. 5b & c; *p* < 0.05). In the absence of NPY, PD98059 and U0126 had no significant effect on Ki67-positivity (Fig. 5b & c) either trypan blue positivity (data not shown). These results indicate NPY regulates early antral granulosa cell proliferation through NPY5R and MEK signaling pathway.

## Discussion

The molecular and cellular regulation of folliculogenesis is complex and not fully understood. NPY is known to regulate female reproductive function through the central neuron system [6]; NPY at the hypothalamus modulates the activity of GnRH neuronal system via NPY1R and NPY2R and regulates LH secretion by the pituitary gland [15–21]. However, its role in the control of the follicular development at the ovarian level has not been reported. In the present studies, we have demonstrated that: a) the expression of NPY and its receptors is follicular stage-dependent and cell type-specific; b) the influence of NPY on granulosa cell proliferation is different between EA and LAF; and c) NPY-induced granulosa cell proliferation was sensitive to NPY5R antagonist and MEK inhibitors, suggesting that NPY5R signaling may be an intra-ovarian regulatory event in folliculogenesis. These findings support the hypothesis that NPY promotes follicular survival and growth in EA but induces follicular atresia in LAF.

The expression of NPY and its receptors NPY2R in the ovary has been reported [8, 22]. However, whether these responses vary among follicular stages and if granulosa cells are the predominant source of follicular NPY remain to be determined. Our immunofluorescence results suggest that antral granulosa cells are the main follicle source of NPY with the highest expression in EA compared to LAF. However, our western blotting results show no difference of cellular NPY abundance between PA and EA. The reason for this apparent dichotomy is not known, but could be technical differences including purity of the isolated cells (e.g. mixture of antral- and mural-granulosa cells) and assay sensitivity (real-time PCR and Western blot). Regarding NPY receptors, we have found that granulosa cells express all NPY receptors, but their cellular levels vary among follicular stages. In contrast to NPY1R, NPY2R and NPY4R receptors, NPY5R is significantly higher in EA granulosa cells compared to LAF. Our results indicate that function of NPY/NPY5R in granulosa cells varies among follicular stage and its response is strong at early antral stage.

Conflicting findings exist regarding the role of NPY in the regulation of cell proliferation; whereas NPY promotes proliferation in vascular smooth muscle cells [23], bone marrow stromal cells [24] and adipocyte precursor cells [7], it suppresses proliferation in luteinized porcine granulosa cells [14]. Moreover, whether the regulation of granulosa cell proliferation by NPY is follicular stage-dependent is not known. In the present studies, we have demonstrated that NPY at physiological concentration (0.1 nM) [25, 26] induces proliferation in granulosa cells from EA, but not LAF in vitro. Additionally, an antagonist of NPY5R blocked NPY-induced granulosa cell proliferation in EA, as also demonstrated in neuroblastoma [27] and breast cancer [28, 29] cells. Although granulosa cells of PA and EA contain similar NPY5R protein contents, their responses are different. It is indicated that other NPY receptors may have the complementary or compensatory mechanism. Our results suggest that NPY/NPY5R regulates granulosa cell proliferation in a follicular stage-dependent manner, with an induction at EA and suppression at LAF.

Depending on the NPY receptor subtypes and the target cells, various signaling pathways appear to be involved in the regulation of cell proliferation. NPY1R has been reported to enhance proliferation via Wnt/β-catenin pathway in bone marrow stromal cells [30] and MEK signal in adipocyte precursor cells [7]. NPY5R is known to induce proliferation via MEK in cardiomyocytes [31], bone marrow stromal cells [24] and breast cancer cells [28], and STAT3 in vascular smooth muscle cells [23]. It has been reported that MEK and AKT signal pathways were important of proliferation in granulosa cells [32, 33]. Support for this notion came from the observation that the MEK inhibitor U0126 attenuates hepatocyte growth factor-induced proliferation in human granulosa-like tumor cell line (KGN cells) [32] and insulin-like growth factor 1-induced, AKT-mediated proliferation in human luteinized granulosa cells [33]. In the present studies, we have demonstrated that NPY-induced proliferation in granulosa cells may involve NPY5R and the MEK signal pathway. Although NPY5R-mutant female mice are fertile and NPY has been suggested to be a factor which regulates granulosa cell proliferation and follicle selection [34].

## Conclusions

We have demonstrated for the first time the possible role of NPY on ovarian folliculogenesis. We propose the following hypothesis (Fig. 6): during the preantral follicle development, granulosa cells express minimal NPY and NPY5R, and NPY does not affect proliferation nor apoptosis. As the follicle emerges in the early antral stage, granulosa cells express a greater amount of NPY, which induces the proliferation of granulosa cells in an autocrine and a NPY5R-dependent manner. However, during late antral development, NPY and NPY5R expression in granulosa cells decreases, resulting in reduction of proliferation and induction of apoptosis. Whether NPY plays a role in pathogenesis of follicular developmental disorders (e.g. polycystic ovarian syndrome) remains to be determined.

## Methods

### Animal

Female SD rats were obtained from Charles River Canada (Montreal, QC, Canada). All animal procedures were carried out in accordance with the Guidelines for the Care and Use of Laboratory Animals and the Canadian Council on Animal Care, and approved by the University of Ottawa Animal Care Committee (Protocol # OHRI-1624).

### Reagents and materials

Folligon (eCG, equine Chorionic Gonadotropin) was purchased from Merck (Kirkland, Canada). RNeasy minikit and primers were purchased from QIAGEN (Hilden, Germany). SYBER Green I Master, cOmplete (a proteinase inhibitor), PhosSTOP (a phosphatase inhibitor cocktail) and In Situ Cell Death Detection Kit were obtained from Roche Diagnostics GmbH (Mannheim, Germany). Rat Neuropeptide Y was purchased from Abcam (Cambridge, MA). Bovine serum albumin (BSA), paraformaldehyde (PFA), phosphate-buffered saline containing 0.05% tween-20 (PBS-T) and 5-bromo-2-deoxyuridine (BrdU) were purchased from Millipore Sigma (Oakville, Canada). Six-well plate and 8 chamber slides were from Corning (Corning, NY). High-Capacity cDNA Reverse Transcription Kits, M199, penicillin and streptomycin, amphotericin B, fetal bovine serum (FBS) and SlowFade™ Gold Antifade Mountant with DAPI were purchased from Thermo Fisher Scientific (Waltham, MA). Sodium citrate was purchased from Fisher Scientific (Nepean, Canada). Cell lysis buffer was purchased from Cell signaling technology (Danvers, MA). Bio-Rad DC Protein Assay Reagent was from Bio-Rad Laboratories (Hercules, CA). The antibodies used in this study are described in Additional file 1: Table S1.

### Immunofluorescence

For immunofluorescence of NPY, whole ovaries from 21-day-old immature rats were fixed in 4% PFA (24 h, 4 °C) and embedded in paraffin. Thirty serial sections (Thickness: 5 μm) were cut from each sample to attain 150 μm of distance between analyzed sections and to avoid the double analysis of same follicle [35]. Ovarian sections were deparaffinized and hydrated. Antigen retrieval was achieved with citrate buffer (10 mM; pH 6.0, boiled by microwave, 1 min). Sections were washed in PBS-T and non-specific bindings were blocked with 5% skim milk [40 min, room temperature (RT)]. The slides were incubated with rabbit anti-NPY or rabbit IgG, polyclonal (isotype control; overnight, 4 °C) followed by anti-rabbit conjugated with alexa-fluor 594 (1 h, RT). Ovarian sections were mounted with SlowFade™ Gold Antifade Mounting with DAPI, and images were observed by fluorescence microcopy (Zeiss Axioplan 2, Zeiss, North York, Canada), recorded with the Axion Vision program (Axion Vision software, Zeiss) and analyzed using FIJI software.

Immunofluorescence of Ki67 was performed to assess granulosa cell proliferation [36]. Cells were fixed with 4% PFA (60 min, RT) and treated with 0.25% Triton-100 (3 min, RT). Nonspecific binding was blocked with 3% BSA (30 min, RT). Cells were incubated (overnight, 4 °C) with anti-Ki67 antibody or rabbit IgG, polyclonal (isotype control) followed by anti-rabbit IgG conjugated with alexa-fluor 594 (1 h, RT). Cells were washed, mounted with SlowFade™ Gold Antifade Mountant with DAPI and observed by fluorescence microscope with images recorded using the Axion Vision program. At least 400 cells were observed per experimental group.

### Granulosa cell isolation

Granulosa cells from follicles at different stages of development [37] were isolated as previously described [38]. Briefly, ovaries from 21-day-old rats injected with eCG (10 IU, ip) were collected at various period after injection and kept in M199: 0 h (predominantly PA and EA), 24 h (predominantly EA), and 48 h (predominantly LAF and preovulatory follicles). Granulosa cells were plated overnight (0.5 × 10^6^ per well in 6 well plate; 35,000 per well in chamber slide) in M199 containing penicillin, streptomycin and amphotericin B with 10% FBS under a humidified atmosphere of 95% air and 5% CO_2_. After a 24-h culture in serum-reduced medium, granulosa cells were treated with NPY for 24 h for assessment of Ki67 positivity, TUNEL and BrdU assays.

### Western blotting

Granulosa cells were lysed in cell lysis buffer containing cOmplete and PhosSTOP, a protease inhibitor and phosphatase inhibitor cocktail. Following sonication, the lysates were centrifuged (20 min, 15,000 g, 4 °C) and protein concentration was determined (Bio-Rad DC Protein Assay Reagent). To analyze NPY and NPY receptors contents, 40 μg and 20 μg protein lysates were separated by 16.5% Tricine-SDS-PAGE and 10% SDS-PAGE, respectively. Separated proteins were electro-transferred to PVDF membranes (for NPY) and nitrocellulose membranes (for NPY receptors). Nonspecific bindings to the membranes were blocked with skim milk (5%, 1 h, RT). Blots were incubated with primary antibody (overnight; 4 °C) and then with HRP-conjugated secondary antibody (1 h, RT). Peroxidase activity was visualized with an enhanced chemiluminescence kit, and membranes were exposed to X-ray film. Signals were densitometrically quantified using FIJI software.

### RNA extraction, reverse transcription and real-time PCR

RNA was extracted from granulosa cells by RNeasy minikit. One μg of total RNA was reverse transcribed in a 20-μl of a reaction, using a High Capacity cDNA Reverse Transcription Kit. Real-time PCR was run using Light Cycler® 480 SYBR Green I (Roche Diagnostics GmbH) with the following conditions: Npy, 45 cycles at 95 °C (10 s), 60 °C (10 s), 72 °C (7 s); Gapdh, 45 cycles at 95 °C (10 s), 60 °C (10 s), 72 °C (7 s). Values were calculated by subtracting data for Ct values of the internal standard (glyceraldehyde dehydrogenase, Gapdh) from Ct values of Npy. Data were analyzed by the 2-ΔΔCT method.

### Apoptosis assay

Apoptosis was assessed by the TUNEL assay, using the In Situ Cell Death Detection Kit. Stained cells were observed by fluorescence microcopy and images were recorded using the Axion Vision program. At least 400 cells were observed per experimental group.

### BrdU (5-bromo-2-deoxyuridine) incorporation

Granulosa cells were incubated with BrdU (10 μM, Millipore Sigma) for 4 h, fixed with 70% ethanol and kept at − 20 C° overnight. They were incubated with 2 N HCl/0.5%TritonX-100 (30 min, RT) to produce single-stranded DNA and neutralized with 0.1 M Sodium tetraborate decahydrate (2 min, RT), and stained with anti-BrdU-FITC antibody (30 min, RT). Flow cytometry acquisition was performed using BD LSRFortessaTM (BD, Franklin, NJ) and the data were analyzed using the FlowJo® software. Doublets were excluded with forward scatter height against forward scatter area and subsequently side scatter height against side scatter area. Granulosa cells incubated without BrdU were used as a negative control.

### Statistical analysis

Results are expressed as mean ± SEM and analyzed by paired *t* test, unpaired *t* test or one- or two-way ANOVA, with Tukey’s post hoc analysis for multiple comparisons. Statistical analyses were performed using Prism 7 (GraphPad software Inc.). Significant differences were considered at *P* < 0.05.

## Supplementary information

**Additional file 1: Table S1.** Antibodies used in the present studies.

## Data Availability

All data is contained in the manuscript.

## Abbreviations

BAX: Bcl-2-associated X
BrdU: 5-bromo-2-deoxyuridine
BSA: Bovine serum albumin
EA: Early antral follicles
eCG: Equine chorionic gonadotropin
FBS: Fetal bovine serum
LAF: Late antral follicles
MEK: Mitogen-activated protein kinase
NPY: Neuropepetide Y
NPY1R: NPY receptor Y1
NPY2R: NPY receptor Y2
NPY4R: NPY receptor Y4
NPY5R: NPY receptor Y5
PA: Preantral follicles
PBS-T: Phosphate-buffered saline containing 0.05% tween-20
PCNA: Proliferating cell nuclear antigen
PCR: Polymerase chain reaction
PFA: Paraformaldehyde
RT: Room temperature
SD rat: Sprague Dawley rat
