# Supplementary material for: Neuropeptide Y regulates proliferation and apoptosis in granulosa cells in a follicular stage-dependent manner
Source: J Ovarian Res. 2020 Jan 8;13:5. doi: 10.1186/s13048-019-0608-z (PMC6950994; doi:10.1186/s13048-019-0608-z)
Supplement: Supplementary file 1 — Additional file 1: Table S1. Antibodies used in the present studies. [file 13048_2019_608_MOESM1_ESM.docx]

**Table S1: Antibodies used in the present studies**

| Product name | Source  (Company) | Catelogue number | Dilution in specific application | | | |
| --- | --- | --- | --- | --- | --- | --- |
|  |  |  | WB | IHC | ICC | FCM |
| Neuropeptide Y (D7Y5A) XP® Rabbit mAb | Cell Signaling | 11976 | 1:1000 |  |  |  |
| anti-Neuropeptirde Y antibody | Abcam | ab30914 |  | 1:500 |  |  |
| Anti-Ki67 antibody | Abcam | ab15580 |  |  | 1:100 |  |
| Anti-NPY1R antibody | Abcam | ab216966 | 1:500 |  |  |  |
| Anti-Neuropeptide Y2 Receptor | Alomone labs | ANR-022 | 1:500 |  |  |  |
| Anti-Neuropeptide Y4 Receptor | Alomone labs | ANR-024 | 1:500 |  |  |  |
| Anti-NPY5R antibody | Abcam | ab133757 | 1:15000 |  |  |  |
| Anti-GAPDH antibody | Abcam | ab181602 | 1:3000 |  |  |  |
| Rabbit IgG, Polyclonal-Isotype Control | Abcam | ab171870 |  |  |  |  |
| Goat Anti-Rabbit IgG (H+L)-HRP Conjugate | Bio-Rad | 170-6515 | 1:2000 |  |  |  |
| Goat Anti-Mouse IgG (H+L)-HRP Conjugate | Bio-Rad | 170-6516 | 1:2000 |  |  |  |
| Goat anti-Rabbit IgG (H+L) Cross-Adsorbed Secondary Antibody, Alexa Fluor 594 | Thermo Fisher Scientific | A-11012 |  | 1:200 | 1:200 |  |
| BrdU Monoclinal antibody (BU20A), FITC | Life technology | 11-5071-41 |  |  |  | 1:20 |
| Ki-67 Monoclonal Antibody (SolA15), FITC | Life technology | 11-5698-82 |  |  |  | 1:200 |
| Rat IgG2a kappa Isotype Control (eBR2a), FITC | Life technology | 11-4321-80 |  |  |  |  |

WB: Western blot;

IHC: Immunohistochemistry;

ICC: Immunocytochemistry;

FCM: Flow cytometry
